# Cellular senescence-related gene variants as risk factors for recurrent pregnancy loss

**DOI:** 10.1007/s11033-026-12281-0

**Published:** 2026-07-28

**Authors:** Eduarda Nabinger, Luiza Pretto, Eduardo Cremonese Filippi-Chiela, Thayne Woycinck Kowalski, Maria Teresa Vieira Sanseverino, Fernanda Sales Luiz Vianna, Lucas Rosa Fraga

**Affiliations:** 1 Graduate Program in Medicine: Medical Sciences, School of Medicine, Federal University of Rio Grande do Sul, Porto Alegre, Brazil; 2 Experimental Research Center, Hospital de Clínicas de Porto Alegre, Porto Alegre, Brazil; 3 Department of Morphological Science, Institute of Health Sciences, Federal University of Rio Grande do Sul, Porto Alegre, Brazil; 4 Medical Genetics Service, Hospital de Clínicas de Porto Alegre, Porto Alegre, Brazil; 5 Graduate Program in Genetics and Molecular Biology, Department of Genetics, Biosciences Institute, Federal University of Rio Grande do Sul, Porto Alegre, Brazil

**Keywords:** Abortion, Miscarriage, Cell Aging, Infertility, Polymorphism

## Abstract

**Background:**

Recurrent pregnancy loss (RPL) is characterized by two or more pregnancy losses. Despite its etiology encompassing known risk factors, over half of the cases remain idiopathic. In this sense, the search for molecular and cellular mechanisms that could be related to this condition is essential for explaining, at least in part, these cases. Cellular senescence (CS) is a state of cell cycle arrest that is present during reproduction and development; however, its impact on pregnancy remains unclear. Thus, our purpose was to evaluate the involvement of CS-related genes in the RPL context.

**Methods and Results:**

First, differential gene expression (DGE) of *CDKN1A*, *CDKN2A*, *AKT1*, *EP300*, *TNF*, and *IFNG* was assessed through a secondary analysis of publicly available datasets in the placenta and endometrium of RPL patients. Then, genetic variants (rs2395655, rs11515, rs1130233, rs20551, rs361525, and rs2430561, respectively) of these genes were evaluated in 107 women with RPL and 118 fertile controls. No difference in the expression of the genes assessed was found between the groups. On the other hand, the frequency of the *CDKN1A* allele A rs2395655 was more present in RPL patients than in fertile controls. In addition, a multiple comparison revealed two genotype combinations that were differently distributed between the groups (rs2395655 AG and rs11515 GG were more frequent in the RPL group; rs2395655 AG and rs11515 CG were more frequent in the controls), which might indicate them as associated with increased and decreased susceptibility factors, respectively, for RPL.

**Conclusion:**

Although our data are preliminary, we hypothesize that CS may represent a fraction of RPL cases.

**Supplementary Information:**

The online version contains supplementary material available at 10.1007/s11033-026-12281-0.

## Introduction

Recurrent pregnancy loss (RPL) is characterized by the loss of two or more pregnancies and affects around 1–2% of couples, as defined by the European Society of Human Reproduction and Embryology (ESHRE) [1]. The etiology of RPL encompasses many known risk factors, including the presence of chromosomal abnormalities in the couple, female anatomical anomalies, infections, autoimmune diseases, and endocrine disorders [2]. Although many causes have been described for RPL, in about half of the cases, the etiology remains unknown, making it necessary to conduct a more indepth investigation of the factors that might, at least in part, explain the occurrence of such unsolved episodes. Therefore, it is essential to seek mechanisms involved with pregnancy that could be associated with RPL.

Cellular senescence (CS), in turn, is the state in which cells are no longer able to divide even after being stimulated with growth factors [3]. CS may be the result of telomere shortening (replicative senescence) or cellular stress (induced senescence), with the latter activated by a range of intrinsic and extrinsic cell factors – including oncogenic activation, oxidative and genotoxic stresses, mitochondrial dysfunction, irradiation, and action of chemotherapeutic agents [4–6]. These cells present higher lysosomal content and express a characteristic secretory phenotype, named Senescence-Associated Secretory Phenotype (SASP) [7–8]. CS is involved in several physiological contexts, including tumor suppression, wound regeneration, and plays an essential role during embryonic and fetal development, being present in embryonic signaling centers and contributing to the specification of cell fates and tissue patterning [9–10].

Senescent cells are found in well-established time and space, performing functions and subsequently disappearing, demonstrating that their induction, presence, and removal are a programmed and tightly controlled cellular process [11]. In addition, SASP factors secreted by senescent cells share common markers with the profile of molecules secreted by gestational tissues. Thus, SASP components seem to play an important role during embryo development by creating an environment that promotes placental development and vascular remodeling [12]. Some molecular markers have been studied previously in relation to cellular senescence and reproduction. *CDKN1A*, located on chromosome 6, which encodes protein p21; and *CDKN2A*, located on chromosome 9, which encodes proteins p14Arf and p16, are key genes that respond to stress, establishing and maintaining cell cycle arrest [13–15]. Other genes, such as *AKT1* (chromosome 14) and *EP300* (chromosome 22) and their encoded proteins also play an underlying role in CS through the regulation of cell growth and proliferation, differentiation, and cell survival [16–17]. Tumor necrosis factor (TNF)-α and interferon gamma (IFN)-γ, encoded by their respective genes, which are located on the 6 and 12 chromosomes, respectively, are two SASP cytokines that have a regulatory role during pregnancy. TNF-α is related to trophoblast invasion and labor, whereas IFN-γ is associated with vascular remodeling, implantation, and placenta formation [18–20].

Whereas many advances have been made to unravel the causes and risk factors for RPL, the high number of idiopathic cases makes the continuous investigation in this field necessary. CS has been increasingly studied in recent years, and in terms of reproduction and development, it is related to physiological processes. In maternal-fetal tissues, CS is associated with human pregnancy and parturition [21]. However, CS deregulation was associated with pregnancy and embryonic/fetal development complications, such as RPL [21]. Thus, given CS complexity and relationship with reproduction, it appears to be a relevant mechanism with a potential role in RPL. Therefore, the aim of this study was to evaluate the involvement of CS in RPL through differential gene expression analysis and genetic variants evaluation of *CDKN1A*, *CDKN2A*, *AKT1*, *EP300*, *TNF*, and *IFNG* genes in RPL and control patients.

## Materials and methods

This study was conducted following the recommendations of the STROBE (Strengthening the Reporting of Observational studies in Epidemiology) initiative [22] and was approved by the Research Ethics Committee of Hospital de Clínicas de Porto Alegre (registration number 2022 − 0153; CAAE number 58396022.0.0000.5327). All women provided written informed consent in accordance with the Declaration of Helsinki. The data presented here were partially published in conference proceedings in abstract form [23].

### Gene expression analysis

*CDKN1A*, *CDKN2A*, *AKT1*, *EP300*, *TNF*, and *IFNG* genes had their expression evaluated using publicly available data obtained from three different studies deposited as datasets on Gene Expression Omnibus (GEO) repository [24], as previously reported by Bremm et al. (2021) [25], through a literature-based in silico approach. Datasets were identified through systematic searches in the GEO database using the keywords “miscarriage” and “pregnancy loss,” restricted to *Homo sapiens* and “expression profiling by array”, followed by manual screening based on relevance to endometrial, placental, or reproductive tissue expression in pregnancy loss-related conditions. Differential gene expression (DGE) was evaluated in two distinct reproductive tissues from women with RPL and fertile women: (1) placenta (GSE22490) [26]; and (2) endometrium (GSE26787 and GSE165004) [27–28]. Microarray .CEL data were downloaded from GEO, and robust multi-averaging (RMA) normalization was performed using the Affy package [29] in R Studio v. 4.2.0. Probes were accessed according to the JetSet algorithm [30].

### Sample

A total of 107 women who were under investigation for RPL (RPL group) referred to the Prenatal Diagnosis and Reproductive Genetics Ambulatory of the Medical Genetics Service (DPN-SGM) at the Hospital de Clínicas de Porto Alegre (HCPA) between 2000 and 2011, and 118 women with no history of pregnancy loss or infertility and with at least two alive children (controls) were included by convenience in this case-control study. Both groups are part of a sample studied by Fortis et al., 2018 [31], where a more detailed description of subjects’ selection can be found. Clinical and epidemiological data, as well as risk factors for RPL, were evaluated and compared in both groups. Investigations for possible RPL causes included: anatomical defects, hormonal status, chromosomal abnormalities, immunological risk factors, acquired thrombophilia (anti-phospholipid antibodies), and inherited thrombophilia (Factor V-Leiden, Factor II G20210A) [32, 33]. Not all women underwent all tests; however, when a cause that explained the RPL was identified, the patient was withdrawn from the study.

### Variants selection and genotyping

A search for gene variants was performed from the public databases SNP (NCBI), Ensembl [34], ABraOM [35], and PubMed for *CDKN1A*, *CDKN2A*,* AKT1*, *EP300*, *TNF*, and *IFNG*. Variants with global population frequency higher than 5% were considered, and the choice took into account whether the listed genetic variant had already been previously studied in any clinical condition. Sample collection and DNA extraction were performed by the Oragene^®^ kit for the collection and extraction of salivary DNA (DNA Genotek Inc., Canada) or the Puregene^®^ kit for blood DNA extraction (QIAGEN, Inc., Germany), according to the manufacturer’s instructions. Genotypic determination was performed using the following TaqMan^®^ Genotyping Assays (Applied Biosystems, USA): C__16223289_10 for variant rs2395655 (*CDKN1A*); C__12096259_10 for variant rs11515 (*CDKN2A*); C___7489835_10 for variant rs1130233 (*AKT1*); C___2519323_1 for variant rs20551 (*EP300*); C__2215707_10 for variant rs361525 (*TNF*); and a customized TaqMan SNP genotyping assay for variant rs2430561 (*IFNG*; F: 5′-TTCAGACATTCACAATTGATTTTATTCT-3′ and R: 5′-CCCCCAATGGTACAGGTTTC-3′; and probes (FAM/VIC: AAAATCAAATC[T/A]CACACACACA-MGB) as previously described by Tso et al. (2005) [36]. Real-time PCR was conducted according to the manufacturer’s instructions with Fast Advanced Master Mix in a protocol adapted for a reaction with a final volume of 12 µL in the QuantStudio3 real-time PCR system (Thermo Fisher, USA). The results were analyzed using QuantStudio Design & Analysis v.2.6.2 software.

### Statistical analysis

For gene expression analysis, the Shapiro-Wilk test was performed to evaluate data normality, and the *t*-Test or the Mann-Whitney U test to compare RPL *versus* fertile women. For the case-control analysis, sample characterization, and clinical and demographic analysis, Chi-square and Mann-Whitney tests were performed in SPSS v.18.0 software (IBM Corp., USA). To verify the Hardy-Weinberg equilibrium, a Chi-square test was performed in BioEstat v.5.3 software (Instituto Mamirauá, Brazil). Chi-square test was also performed in WINPEPI v.11.65 software [37] and SPSS v.18.0 software (IBM Corp., USA) to compare allelic and genotypic frequencies, respectively. In addition, Chi-square with Z test and Bonferroni correction was performed in SPSS v.18.0 software (IBM Corp., USA) to evaluate the combination of genotypes by a multiple comparison analysis. Logistic regression analyses were conducted using SPSS v.18.0 software (IBM Corp., USA). Odds ratios (ORs) and 95% confidence intervals were estimated for the evaluated combinations and adjusted for potential confounders, including age and alcohol consumption. In the absence of a priori sample size calculation, post hoc power analyses were performed using PSS Health (Power and Sample Size for Health Researchers, Brazil) to estimate the minimum detectable OR for each genetic combination, considering the observed sample size and a significance level of 0.05. For all analyses, *p*-values < 0.05 were considered statistically significant.

## Results

**Fig. 1:**
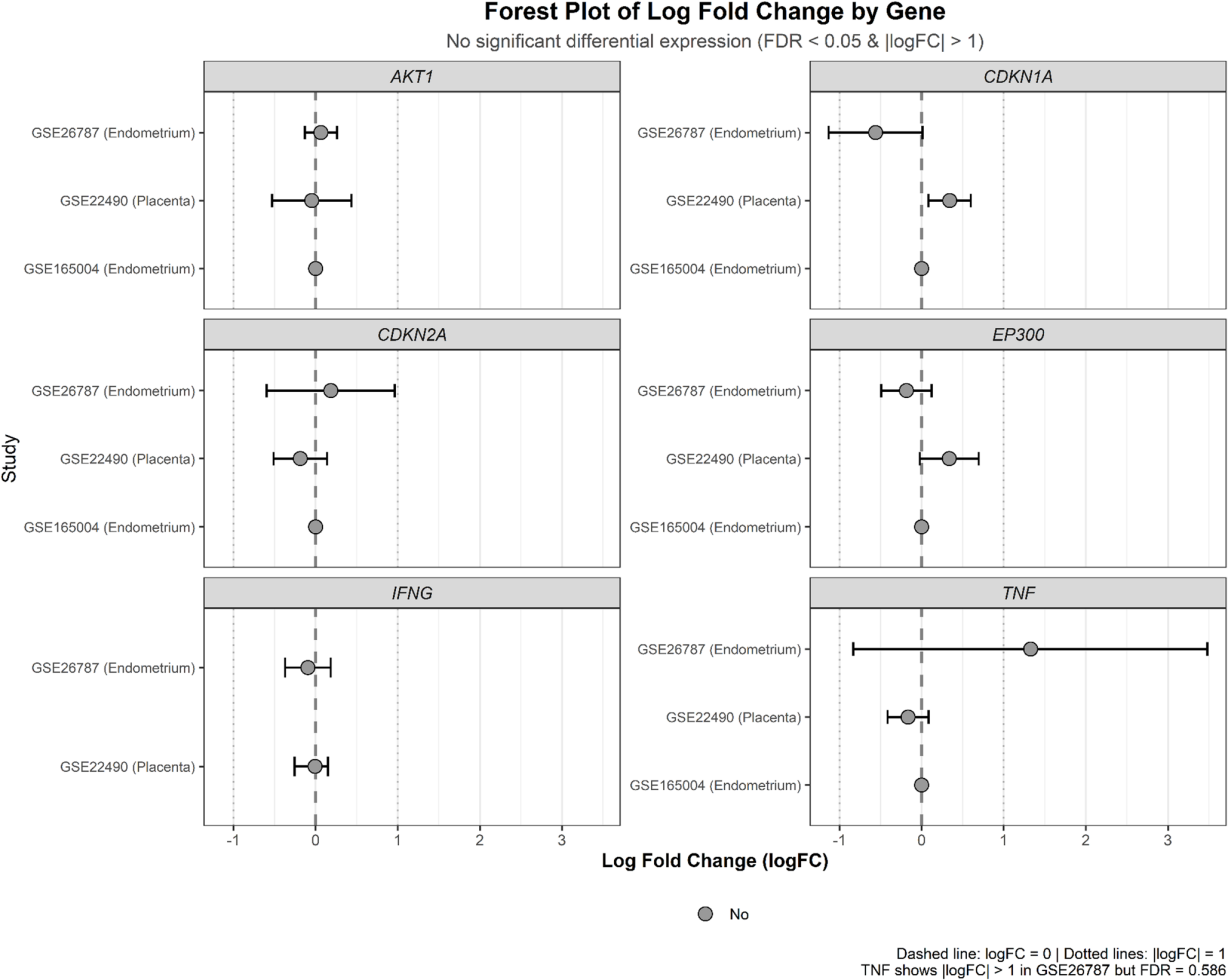
Forest plot of log fold change by each gene analyzed in the study. No significant expression was found (FDR < 0.05 & |logFC| > 1). Dashed line: logFC = 0 | Dotted lines: |logFC| = 1 TNF shows |logFC| > 1 in GSE26787 but FDR = 0.586

It is known that CS is related to human development and reproduction. Still, the role of this cellular process in adverse reproductive outcomes such as RPL is not well understood. Trying to explore the role of CS on RPL, differential gene expression (DGE) analysis of *CDKN1A*, *CDKN2A*, *AKT1*, *EP300*, *TNF*, and *IFNG* was conducted through a secondary analysis of publicly available datasets, comparing women who experienced RPL (case group) and fertile women (control group) in placental and endometrial tissues using data from GEO repository. However, a statistically significant difference in the expression patterns of the analyzed genes in both tissues between the two groups was not found (Supplemental Table 1). In addition, a forest plot with the logFC and *p*-values adjusted by false discovery rate (FDR) is available in Fig. 1. Still, the GSE165004 dataset had no probe for *IFNG*.

Next, with the aim of further investigating the role of CS in RPL, a genetic analysis was performed through a case-control study to evaluate if the variants in *CDKN1A* (rs2395655), *CDKN2A* (rs11515), *AKT1* (rs1130233), *EP300* (rs20551), *TNF* (rs361525) and *IFNG* (rs2430561), important molecular markers to CS, could be related to genetic susceptibility to RPL. Regarding demographic and clinical data, the median age at recruitment between the groups under study differed (*p* < 0.001), however the age during first pregnancy did not (Table 1). Couple consanguinity and alcohol consumption were higher in the RPL group (*p* < 0.001; Table 1) than in controls. Other variables, such as the number of pregnancies, smoking, and body mass index (BMI), and self-declared ancestry, were not significantly different between the groups. Given the use of DNA samples from a biorepository previously collected, some demographic and clinical information was not available in medical records for a subset of individuals; however, all analyses were conducted using the available data for each variable, and this limitation did not affect the main findings of the study.

**Table 1:** Demographic and clinical data of RPL and control groups

Characteristic	RPL group	Control group	*p* value
Frequency [N(%)]	107 (47.56)	118 (52.44)	
Age [median (IQR)]	32.8 (28–37)	45 (37–52)	< 0.001ᵃ
Age of first pregnancy [median (IQR)]	24 (18–28)ᶜ	23 (20–25)ᵈ	0.721ᵃ
Pregnancies [mean (SD)]	3 (1.70)	2.98 (1.32)	0.410ᵃ
Miscarriages [mean (SD)]	3 (1.50)	N/A	
Couple consanguinity [N(%)]	5 (5.95)ᵉ	0ᶠ	< 0.001ᵇ
Smoking [N(%)]	17 (15.89)	25 (21.55)ᵍ	0.127ᵇ
Occasional alcohol consumption [N(%)]	39 (36.45)	20 (17.24)ᵍ	< 0.001ᵇ
Body mass index (BMI) [mean (SD)]	25.80 (4.32)ᵈ	23.77 (4.34)ʰ	0.131ᵃ
Ancestry [N(%)]	^i^	^j^	
European ancestry [N(%)]	77 (74.8)	100 (85.5)	0.129^a^
African ancestry [N(%)]	15 (14.6)	11 (9.4)	
Other ancestries [N(%)]	11 (10.7)	6 (5.1)	

RPL: recurrent pregnancy loss; N/A: not applicable; N: number; IQR: interquartile range; SD: standard deviation

ᵃMann-Whitney U-test; ᵇPearson Chi-Square test; ᶜN= 105; ᵈN= 107; ᵉN= 84; ᶠN= 115; ᵍN=116; ʰN=36

Regarding genotyping, all genotypic and allelic frequencies were within the range expected by the Hardy-Weinberg distribution. The genotypic and allelic frequencies of the genes under study did not differ between cases and controls, except for the allele frequency of *CDKN1A* (Table 2). The allele A frequency of *CDKN1A* was found to be more present in RPL patients (*p* = 0.001; Table 2), different from the other variants, of which the genotypic and allelic frequencies did not differ between cases and controls. Considering the close functional relationship between the proteins encoded by the *CDKN1A* and *CDKN2A* genes, together with the statistically significant association observed for the allelic frequency of *CDKN1A*, we subsequently evaluated the combined effects of the genotypes of the variants analyzed in these genes by a multiple comparison analysis. It was found that *CDKN1A* AG and *CDKN2A* GG genotypes were more frequent in the RPL group, whereas *CDKN1A* AG and *CDKN2A* CG genotypes were more frequent in controls (Table 3). These data suggest that the effect of the combination is related to the genotype of *CDKN2A*. To confirm this, the frequencies of the genotype combinations *CDKN1A* AG and *CDKN2A* GG and *CDKN1A* AG and *CDKN2A* CG were compared to the other combinations of genotypes present in the analyzed sample. It was observed that the two combinations remained more frequent in the RPL and in the control group, respectively (Table 4). Finally, the odds ratio (OR) of these combinations was estimated, being observed that women with RPL have 87.7% more chance of having *CDKN1A* AG and *CDKN2A* GG genotype combination (OR = 1.877, CI 95%: 1.076–3.303), while those that are not experiencing the condition have 69% more chance of having *CDKN1A* AG and *CDKN2A* CG genotype combination (OR = 0.310, CI 95%: 0.119–0.766). Considering patients’ age and alcohol consumption differed between the groups, which could potentially influence the results, the odds ratios were recalculated and adjusted for these variables. Statistical significance was maintained, with adjusted ORs of 2.150 (1.066–4.438) and 0.246 (0.069–0.763), respectively. These findings are supported by statistical power values of 72.7% and 72.4%, respectively.

**Table 2:** Genotypic and allelic frequencies of *CDKN1A*, *CDKN2A*, *AKT1* and *EP300* variants in women with RPL and in the control group

Gene (Variant)	Genotype/Allele of Variant	RPL group		Control group		*p* value	RPL HWE *p*	Controls HWE *p*
		*N*	(%)	*N*	(%)			
*CDKN1A* (rs2395655)	AA	41	38.32	42	35.60	0.549ᵃ	0.892ᵃ	0.398ᵃ
	GG	15	14.02	23	19.49			
	AG	51	47.66	53	44.91			
	A	133	62.15	107	45.34	0.001ᵇ		
	G	81	37.85	129	54.66			
*CDKN2A* (rs11515)	CC	1	0.93	2	1.69	0.528ᵃ	0.666ᵃ	0.595ᵃ
	GG	83	77.57	84	71.19			
	CG	23	21.50	32	27.12			
	C	25	11.68	36	15.25	0.333ᵇ		
	G	189	88.32	200	84.75			
*AKT1* (rs1130233)	CC	58	54.21	68	57.62	0.874ᵃ	0.576ᵃ	0.425ᵃ
	TT	9	8.41	9	7.63			
	CT	40	37.38	41	34.75			
	C	156	72.90	177	75.00	0.689ᵇ		
	T	58	27.10	59	25.00			
*EP300* (rs20551)	AA	56	52.34	59	50.00	0.939ᵃ	0.342ᵃ	0.381ᵃ
	GG	11	10.28	13	11.02			
	AG	40	37.38	46	38.98			
	A	152	71.03	164	69.49	0.800ᵇ		
	G	62	28.97	72	30.51			
*TNF* (rs361525)	GG	94	87.85	105	88.98	0,837ᶜ	0.503ᵃ	0.526ᵃ
	AA	0	0	0	0			
	AG	13	12.15	13	11.02			
	A	13	6.07	13	5.51	0.956ᵇ		
	G	201	93.93	223	94.49			
*IFNG* (rs2430561)	TT	45	42.06	45	38.13	0.699ᵃ	0.630ᵃ	0.381ᵃ
	AA	15	14.02	21	17.80			
	TA	47	43.92	52	44.07			
	A	77	35.98	94	39.83	0.458ᵇ		
	T	137	64.02	142	60.17			

RPL: recurrent pregnancy loss; HWE: Hardy-Weinberg equilibrium

ᵃ Pearson Chi-Square test; ᵇ Pearson Chi-Square test with Yates correction; ᶜ Fisher's exact test

**Table 3:** *CDKN1A* and *CDKN2A* genotype combinations

Genotype	RPL group		Control group		Global *p* valueᵃ
	*N*	(%)	*N*	(%)	
*CDKN1A* AG and *CDKN2A* GG	44*	41.12	32*	27.12	0.046
*CDKN1A* AG and *CDKN2A* CG	6*	5.61	19*	16.10	
*CDKN1A* AG and *CDKN2A* CC	1	0.93	2	1.69	
*CDKN1A* AA and *CDKN2A* GG	30	28.04	33	27.97	
*CDKN1A* AA and *CDKN2A* CG	11	10.28	9	7.63	
*CDKN1A* GG and *CDKN2A* CG	6	5.61	4	3.39	
*CDKN1A* GG and *CDKN2A* GG	9	8.41	19	16.10	
Total	107	100	118	100	

RPL: recurrent pregnancy loss

* : significant difference between groups

ᵃ Pearson Chi-Square test with Z test and Bonferroni correction

**Table 4:** *CDKN1A* AG and *CDKN2A* GG and *CDKN1A* AG and *CDKN2A* CG genotype combinations against the remaining genotype combinations

Genotype	RPL group		Control group		Crude OR (CI)	*p* valueᵃ	Adjusted OR (CI)^b^	*p* valueᵃ
	*N*	(%)	*N*	(%)				
*CDKN1A* AG and *CDKN2A* GG	44	41.12	32	27.12	1.877 (1.073–3.303)	0.027	2.150^c^ (1.066–4.438)	0.035
Remain genotype combination	63	58.88	86	72.88				
*CDKN1A* AG and *CDKN2A* CG	6	5.60	19	16.10	0.310 (0.109–0.766)	0.017	0.246^d^ (0.069–0.763)	0.021
Remain genotype combination	101	94.40	99	83.90				

RPL: recurrent pregnancy loss; OR: odds ratio; CI: confidence interval

ᵃ Pearson Chi-Square test; b Ajusted for age and alcohol consumption; cThe post-hoc statistical power for the outcome was 72.7%; dThe post-hoc statistical power for the outcome was 74.4%

Collectively, although no consistent differences were observed in gene expression levels between cases and controls, the genetic analysis suggests that specific variants and genotype combinations in core cellular senescence-related genes, particularly involving *CDKN1A* and *CDKN2A*, may be associated with susceptibility to RPL.

## Discussion

Recurrent pregnancy loss (RPL) is a challenge in terms of reproductive medicine, and many efforts are needed to help in understanding its genetic background and the risk factors involved. Previous studies showed a relationship between cellular senescence (CS) and embryo and fetal development, as well as its presence during placental formation and parturition [21]. Given that, in this study, it was explored how CS could be related to RPL through a genetic evaluation. Variants in *CDKN1A*, *CDKN2A*, *AKT1*,* EP300*, *TNF*, and *IFNG* genes were evaluated in a sample of patients who experienced RPL and investigated whether they could be risk factors for RPL by comparing them to fertile controls. Interestingly, the allelic distribution of *CDKN1A* (rs2395655) and genotypic combinations of *CDKN1A* (rs2395655) and *CDKN2A* (rs11515) were differently distributed between the analyzed groups.

**Fig. 2:**
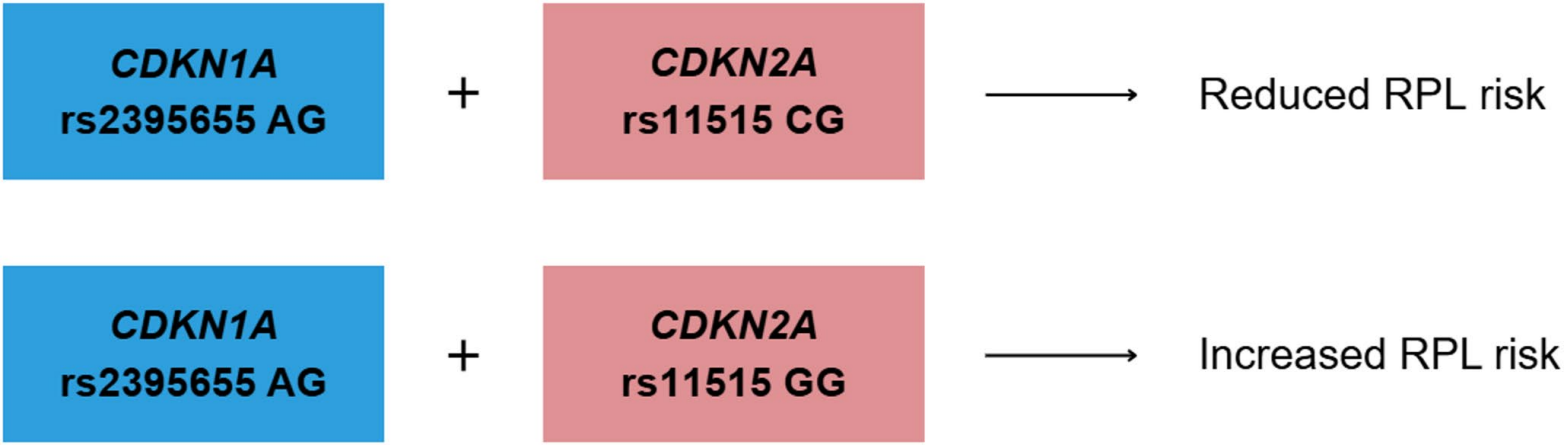
Possible effect of the interaction between the variants rs2395655 AG of *CDKN1A* and rs11515 CG and GG of *CDKN2A* on RPL risk

*CDKN1A* and *CDKN2A* variants were analyzed due to their fundamental role in CS. Several studies have already linked both genes and their encoded proteins to RPL (Table 5). *CDKN1A* expression was increased in chorionic villi, trophoblasts, human decidual cells, and human endometrial stromal cells (hESCs) of women with RPL when compared to control women, as well as *CDKN2A* expression in hESCs from non-receptive patients [38]. Moreover, p21 was associated with trophoblast proliferation and plasticity, and the percentages of p16-positive glandular and luminal epithelial endometrial cells were significantly higher in patients with live births compared with women with pregnancy losses [39]. Although previous studies have demonstrated the involvement of *CDKN1A* and *CDKN2A* in RPL through altered gene and protein expression (Table 5), such investigations focused exclusively on functional and expression-related aspects. To the best of our knowledge, no previous studies have evaluated whether genetic variants in *CDKN1A* and *CDKN2A* contribute to susceptibility to RPL. Therefore, our study addresses an important gap in the literature by investigating the genetic component of two biologically relevant cell cycle and senescence regulators. Our findings point out that the A allele might be implicated in RPL and emphasize that a larger sample size could demonstrate a possible genotypic difference. In addition, it was found that the combination of *CDKN1A* AG and *CDKN2A* GG was more frequent in the case group and the *CDKN1A* AG and *CDKN2A* CG combination was more frequent in the control group. That way, this result suggests that, when combined with the *CDKN1A* AG genotype, the *CDKN2A* rs11515 G allele increases susceptibility to the condition, while the C allele reduces it (Fig. 2). The rs11515 genetic variant is found in an untranslated chromosome region, and some authors suggest it to have an unidentified regulatory function [40]. The *CDKN1A* rs2395655 variant is located in the p53 binding regions and, therefore, can be hypothesized to affect its binding to the *CDKN1A* promoter [41]. On the other hand, the *CDKN2A* rs11515 CG genotype was associated with a decreased expression of the p16 protein, and might act by inhibiting/reducing senescence [42].

**Table 5:** *CDKN1A* (p21) and/or *CDKN2A* (p16 and p14^Arf^) in RPL

CDKN1A (p21) and/or CDKN2A (p14Arf and p16) in Recurrent Pregnancy Loss (RPL)			
Authors	Publication year	Study title	Main findings
Shang et al.	2013	Elevated expressions of *TP53*, *CDKN1A*, and *BAX* in placental villi from patients with recurrent spontaneous abortion	*TP53* expression was significantly increased in chorionic villi of women with RPL compared to control women. In addition, *CDKN1A* and *BAX* mRNA levels as well as the rate of cell apoptosis were elevated in women with RPL.
Lv et al.	2016	Increased apoptosis rate of human decidual cells and cytotrophoblasts in patients with recurrent spontaneous abortion as a result of abnormal expression of *CDKN1A* and *BAX*	The rate of cellular apoptosis and mRNA expression levels of *TP53*, *CDKN1A* and *BAX* were significantly increased in trophoblasts and human decidual cells of women with RPL when compared to control women.
Tomari et al.	2020	Contribution of senescence in human endometrial stromal cells during proliferative phase to embryo receptivity	Human endometrial stromal cells (hESCs) from non-receptive patients exhibited significantly higher proportions of senescent cells, mRNA expression of *CDKN1A* and *CDKN2A* and expression of genes encoding the secretory phenotype associated with senescence (SASP).
Sun et al.	2020	Abnormal Cullin1 neddylation-mediated p21 accumulation participates in the pathogenesis of recurrent spontaneous abortion by regulating trophoblast cell proliferation and differentiation	p21 degradation mediated by neddylated-cullin1 (i.e. a protein related to neddylation process) is required for trophoblast proliferation and may affect trophoblast plasticity.
Parvanov et al.	2021	Decreased number of p16-positive senescent cells in human endometrium as a marker of miscarriage	The percentages of p16-positive glandular and luminal epithelial endometrial cells were significantly higher in patients with live births compared with women with pregnancy losses.

Variants in *AKT1*, *EP300*, *TNF*, and *IFNG* were also investigated. In the reproductive scenario, *AKT1* was already related to deregulation of placental and postnatal growth in mouse models, especially during pre-weaning growth [16–17]. *EP300*, in turn, has been associated with trophoblast differentiation, decidualization, embryo implantation, and preeclampsia [43–44]. Nonetheless, in terms of genetic evaluation, our results did not show a difference in *AKT1* (rs1130233) and *EP300* (rs20551) frequency between the studied groups. Regarding *TNF* and *IFNG* genes, which encode proteins that are part of SASP, TNF-α is associated with term delivery, and the *TNF* rs361525 variant showed a protective effect related to RPL in Italian and Indian women [45–46]. However, in concordance with our findings, no association was found in RPL patients in the Chinese population [47]. IFN-γ is involved with vascular remodeling during the peri-implantation period, early placentation, and the trophoblast invasion process [19]. Higher levels of IFN-γ were reported in RPL patients compared with healthy pregnant women [18, 48], and some studies have shown an association between the *IFNG* rs2430561 variant and RPL, the results are still conflicting, and we were not able to demonstrate a difference in the genetic variant frequency between the RPL group and the control group.

RPL has a multifactorial nature and studies have shown the role of different genetic variants on RPL susceptibility. A recent review by our group showed different genetic variants associated with RPL in the Brazilian population in genes such as *MTHFR*,* FVL*,* FII*, and *IL6* [49]. In addition to individual variant effects, gene-gene interactions may also contribute to RPL; for example, Fraga et al. (2014a, b) found that combinations of *TP53* Arg/Arg (rs1042522) and *MDM2* TT (rs2279744) genotypes, as well as *TP63* TT (rs17506395) and *MDM2* TT (rs2279744), were associated with increased risk of RPL in the Brazilian population [50, 51]. Taken together, these findings, along with the present analysis, highlight the importance of genetic search in RPL. Nonetheless, subsequent studies with increased sample sizes are needed to further evaluate the clinical relevance of these variants and gene-gene interactions as potential markers for RPL.

Based on demographic and clinical characteristics, it was observed that the average age at recruitment of the groups differed, with older controls. This difference is a result of the selection of controls with less risk of becoming pregnant and, consequently, being susceptible to pregnancy loss events, which would result in a bias to the findings. However, there was no difference in the age of first pregnancy, demonstrating the homogeneity between both groups. Alcohol consumption, with higher frequency in the case group, is a well-known risk factor for RPL and was therefore adjusted for in the analyses [52–53]. Importantly, although the post-hoc statistical power for these associations ranged between 72.7% and 74.4%, the p-values reached statistical significance. This suggests that the magnitude of the biological effect of these interactions are robust enough to be detected, despite the limitations inherent in the sample size and the low frequency of certain genotypic combinations in the studied population. However, adjustment for consanguinity - another well-known risk factor for RPL - was not statistically feasible, as no consanguineous couples were present in the control group, resulting in a complete absence of variability for this variable among controls [54].

The DGE analysis using publicly available GEO data did not reveal significant differences. This may be because most samples in the analyzed studies (GSE22490, GSE26787, and GSE165004), as well as in most RPL studies, are collected after pregnancy loss. Such sampling introduces bias, as gene expression at the time might vary between individuals depending on the time required to return to baseline or to establish a new expression pattern, which may differ from that observed during imminent pregnancy loss. Additionally, only the endometrium and placenta were examined, limiting the ability to detect differential gene expression. Thus, DGE analysis is considered tissue- and time-dependent [55–57]. It is important to note that heterogeneity across the analyzed datasets is intrinsic to the original studies, encompassing differences in population characteristics, clinical conditions, and methodological approaches, which may have impacted gene expression variability and the detection of differential expression signals, thereby limiting the findings and their interpretation. Also, gene expression analyses were performed using only publicly available datasets, thereby limiting the incorporation of specific clinical characteristics of our study population. These methodological variables may mask a possible relationship between CS and RPL in our study.

Given the above, our study also has limitations. In addition to those already mentioned, not all women underwent karyotype assessment. This is noteworthy, as chromosomal abnormalities are established risk factors for RPL. However, current international guidelines do not recommend routine parental karyotyping for all couples with RPL. Instead, testing should be considered selectively, particularly in cases where the clinical history suggests a genetic etiology, when there is prior evidence of fetal chromosomal abnormalities, or in the presence of a family history of chromosomal rearrangements [1]. Furthermore, RPL has a complex genetic basis, and variants beyond structural chromosomal rearrangements may also influence pregnancy outcomes, even among couples with identified chromosomal abnormalities [58]. Last but not least, the genetic analysis was limited by a small sample size and by evaluating only one variant per gene, which may have a small effect size on RPL and is insufficient to capture haplotypes effects. We acknowledge that our modest sample size, particularly for genotype combination analyses, may have limited the statistical power to detect smaller effect sizes. Therefore, these findings should be interpreted with caution. However, given the lack of prior studies on *CDKN1A* and *CDKN2A* variants in RPL and the polygenic and multifactorial nature of this condition, we believe these results provide crucial preliminary evidence and help generate hypotheses for future investigations. A larger sample size and the investigation of additional genetic variants, combined with those already studied, might contribute to a better understanding of the relationship between genetic susceptibility and RPL, as well as enable the analysis of possible gene–gene interactions and cumulative genetic effects. Furthermore, these results are based on association analysis and functional studies and genetic analyses in independent cohorts are necessary to validate the proposed mechanisms.

## Conclusion

In summary, although no significant DGE was found, our findings suggest a preliminary association between the *CDKN1A* rs2395655 A allele and RPL, and specific combinations of *CDKN1A* and *CDKN2A* genotypes may be related with the condition. A possible link between *CDKN1A* rs2395655 A allele and *CDKN2A* rs11515 G allele and RPL is also suggested. Importantly, given the multifactorial nature of RPL and the study limitations, these findings are preliminary and should be interpreted with caution, as they are based on association analyses. Therefore, further studies are necessary to confirm the association of variants in *CDKN1A* and *CDKN2A* and RPL.

## Supplementary Information

Below is the link to the electronic supplementary material.

Supplementary Material 1

## Data Availability

The data that support the findings of this study are available from the corresponding author, L.R.F, upon reasonable request.
