# Supplementary material for: Cellular senescence-related gene variants as risk factors for recurrent pregnancy loss
Source: Mol Biol Rep. 2026 Jul 28;53(1):1286. doi: 10.1007/s11033-026-12281-0 (PMC13415376; doi:10.1007/s11033-026-12281-0)
Supplement: Supplementary file 1 — Supplementary Material 1 [file 11033_2026_12281_MOESM1_ESM.docx]

Supplemental Table 1

| Dataset | Tissue | Gene | logFC | CI.L | CI.R | FDR |
| --- | --- | --- | --- | --- | --- | --- |
| GSE22490 | Placenta | CDKN1A | 0.34133 | 0.08370 | 0.59895 | 0.78658 |
| GSE26787 | Endometrium | CDKN1A | -0.56028 | -1.13223 | 0.01167 | 0.33389 |
| GSE165004 | Endometrium | CDKN1A | -0.00003 | -0.00022 | 0.00016 | 0.86137 |
| GSE22490 | Placenta | CDKN2A | -0.18586 | -0.51150 | 0.13976 | 0.94507 |
| GSE26787 | Endometrium | CDKN2A | 0.18502 | -0.59526 | 0.96260 | 0.86733 |
| GSE165004 | Endometrium | CDKN2A | -0.00009 | -0.00028 | 0.00009 | 0.52453 |
| GSE22490 | Placenta | AKT1 | -0.04631 | -0.52941 | 0.43679 | 0.99559 |
| GSE26787 | Endometrium | AKT1 | 0.06402 | -0.13144 | 0.25948 | 0.80690 |
| GSE165004 | Endometrium | AKT1 | 0.00003 | -0.00007 | 0.00013 | 0.73264 |
| GSE22490 | Placenta | EP300 | 0.33734 | -0.02131 | 0.69601 | 0.87216 |
| GSE26787 | Endometrium | EP300 | -0.18443 | -0.49213 | 0.12326 | 0.59537 |
| GSE165004 | Endometrium | EP300 | -0.00016 | -0.00026 | -0.00006 | 0.01764 |
| GSE22490 | Placenta | TNF | -0.16446 | -0.41295 | 0.08402 | 0.92484 |
| GSE26787 | Endometrium | TNF | 1.32775 | -0.83242 | 3.48096 | 0.58569 |
| GSE165004 | Endometrium | TNF | -0.00002 | -0.00020 | 0.00017 | 0.92016 |
| GSE22490 | Placenta | IFNG | -0.00532 | -0.25692 | 0.15037 | 0.98029 |
| GSE26787 | Endometrium | IFNG | -0.09385 | -0.37116 | 0.18344 | 0.79816 |
